# Sulfate-reducing bacteria and methanogens are involved in arsenic methylation and demethylation in paddy soils

**DOI:** 10.1038/s41396-019-0451-7

**Published:** 2019-06-21

**Authors:** Chuan Chen, Lingyan Li, Ke Huang, Jun Zhang, Wan-Ying Xie, Yahai Lu, Xiuzhu Dong, Fang-Jie Zhao

**Affiliations:** 1 State Key Laboratory of Crop Genetics and Germplasm Enhancement, Jiangsu Provincial Key Laboratory for Organic Solid Waste Utilization, College of Resources and Environmental Sciences, Nanjing Agricultural University, 210095 Nanjing, China; 2 State Key Laboratory of Microbial Resources, Institute of Microbiology, Chinese Academy of Sciences, 100101 Beijing, China; 3 College of Urban and Environmental Science, Peking University, 100871 Beijing, China

**Keywords:** Biogeochemistry, Pollution remediation

## Abstract

Microbial arsenic (As) methylation and demethylation are important components of the As biogeochemical cycle. Arsenic methylation is enhanced under flooded conditions in paddy soils, producing mainly phytotoxic dimethylarsenate (DMAs) that can cause rice straighthead disease, a physiological disorder occurring widely in some rice growing regions. The key microbial groups responsible for As methylation and demethylation in paddy soils are unknown. Three paddy soils were incubated under flooded conditions. DMAs initially accumulated in the soil porewater, followed by a rapid disappearance coinciding with the production of methane. The soil from a rice straighthead disease paddy field produced a much larger amount of DMAs than the other two soils. Using metabolic inhibition, quantification of functional gene transcripts, microbial enrichment cultures and ^13^C-labeled DMAs, we show that sulfate-reducing bacteria (SRB) and methanogenic archaea are involved in As methylation and demethylation, respectively, controlling the dynamics of DMAs in paddy soils. We present a model of As biogeochemical cycle in paddy soils, linking the dynamics of changing soil redox potential with arsenite mobilization, arsenite methylation and subsequent demethylation driven by different microbial groups. The model provides a basis for controlling DMAs accumulation and incidence of straighthead disease in rice.

## Introduction

Arsenic (As) is a toxic metalloid widely present in the environment. Humans are exposed to As primarily via drinking water and food, with rice being one of the most important dietary sources of As [1, 2]. Under anoxic conditions in flooded paddy soils, As is mobilized predominantly as arsenite due to reductive dissolution of iron oxyhydroxides, and microbial As methylation is also enhanced [3–5]. The biogeochemical transformation of As in paddy soils and other wetland systems has been investigated intensively. However, aspects of the As biogeochemical cycle, especially As methylation and demethylation, remain poorly understood. Understanding As methylation and demethylation in paddy soils is important for several reasons. First, As methylation can lead to biovolatilization of methylarsine gases, contributing to global As fluxes [6, 7]. Second, As methylation in paddy soil affects As speciation in rice grain and thus the toxicity to humans [8]. Global surveys of polished white rice showed that dimethylarsenate (DMAs) accounts for between 10 and 90% of the total As pool in rice grain [1, 4], but the reasons causing such a large variation are unclear. It has been shown that DMAs in rice is of the soil microbial origin, because rice plants cannot methylate arsenite [9]. Some individual microbial species capable of methylating As have been isolated from paddy soils [10, 11]. However, key microbial groups mediating As methylation and, particularly, those for As demethylation in paddy soil remain unknown. Third, although DMAs is generally assumed to be less toxic to humans than inorganic As (iAs) [4, 12], it is more toxic to plants [13]. In hydroponic experiments, additions of DMAs can induce the symptoms of the straighthead disease, a physiological disorder in rice [14–16], which is characterized by spikelet sterility, distorted husk, unfilled grains, and erect panicles [16–18]. The disease is widespread in many rice growing areas around the world, resulting in substantial yield losses [16–18]. The biogeochemical processes in paddy soils that are conducive to the production of DMAs and the occurrence of rice straighthead disease are poorly understood, hampering the efforts to control this disease [16].

At the biochemical and molecular levels, the mechanism of As methylation is well understood. Arsenite is methylated stepwise to mono-, di- and tri-methylarsenic species in reactions catalyzed by arsenite *S*-adenosylmethionine methyltransferase (ArsM), using *S*-adenosylmethionine as the methyl donor [19, 20]. More than 30,000 *arsM* genes have been deposited in the NCBI database, most of them being of microbial origin with highly diverse sequences. Methylated As species can also be demethylated by some soil microbes [21]. A C·As lyase that catalyzes the demethylation of trivalent monomethylarsenic (MMAs) has been identified in the aerobic bacterium *Bacillus* sp. MD1 [22], but the enzyme cannot demethylate DMAs. There is no knowledge regarding whether and how methylated As species are demethylated in anoxic paddy soils.

The objectives of the present study were to determine the dynamics of methylated As species in paddy soils upon flooding and to identify microbial groups involved in As methylation and demethylation.

## Materials and methods

### Sample collection and analysis

Soils (0–20 cm) were collected from three paddy fields, two at As-contaminated sites in Hunan province (Chenzhou, CZ and Qiyang, QY) and one from an As-uncontaminated site but showing rice straighthead disease (Xinyang, XY, Henan province). Soils were air-dried and disaggregated and subsamples were ground to < 0.149 mm for chemical analyses as described in Supplementary Methods. Rice panicle samples were also collected from the three paddy fields in 2016 for the analysis of As speciation using HPLC-ICP-MS [23].

### Soil incubation experiments

The three soils were incubated under anaerobic conditions to simulate the paddy field conditions. Soil (200 g, < 2 mm) was placed in a 360 mL glass bottle and 200 mL deionized water were added to form a 5 cm layer of standing water above the soil surface. The bottles were sealed and the headspace was purged with N_2_ gas. Depending on the experiments, the treatments included control, additions of molybdate (Mo, 20 mmol kg^−1^) [24], monofluorophosphate (MFP, 500 mmol kg^−1^) [25], or sodium 2-bromoethanesulphonate (BES, 5 mmol kg^−1^) [24], with or without the addition of DMAs at 40 μmol kg^−1^. Each treatment was replicated in three bottles and incubated at 25 °C in the darkness. Soil porewater was collected weekly using a porewater sampler and pH was determined immediately. Portions of the porewater samples were acidified with concentrated  HCl (100:1, v:v)  and filtered through 0.22 μm membrane filters before analysis [26]. Arsenic species were quantified using HPLC-ICP-MS [26]. Nitrate and sulfate concentrations were determined by ion chromatography and soluble Fe by atomic absorption spectroscopy. Methane in the headspace was collected using syringe and determined using gas chromatography (GC). The redox potential of soil was determined at 4 cm below the soil surface using Pt/Ag-AgCl electrodes. An incubation experiment was conducted to determine the mass balance and distribution of As species in the solution, solid and gas phases following the addition of DMAs (see Supplementary Methods).

An incubation experiment was conducted with the addition of ^13^C-labeled DMAs (^13^C 99 atom%, with C in both methyl groups being labeled), which was synthesized enzymatically and purified according to Supplementary Methods. CZ soil (3 g) was weighted into a 15 mL serum tube. ^13^C-DMAs or unlabeled DMAs (as a control) was added to the soil at 13.3 μmol kg^−1^ soil with three replicates. Eight milliliters of deionized water was added to each tube. The tubes were sealed and the headspace was purged with N_2_. The tubes were incubated at 30 °C in the darkness for 14 and 40 days. Arsenic speciation in the soil solution was determined by HPLC-ICP-MS. The concentration of methane in the headspace was determined by GC, and the ^13^C/^12^C isotope ratios of methane and CO_2_ were determined using GC-IRMS (Thermo Fisher Scientific, Germany) [27]. The ^13^C/^12^C isotope ratios were expressed as δ^13^C values (‰) relative to Vienna Pee Dee Belemnite (V-PDB).

### Extraction of DNA and RNA, quantitative PCR, and sequencing

Total DNA and RNA were extracted from soils (after 2 weeks incubation) and from enrichment cultures (see below) using Power Soil DNA or RNA Isolation Kits (MoBIO Laboratories, USA). DNA and RNA concentrations were measured using a NanoDrop 2000C spectrophotometer. cDNA was synthesized by reverse-transcription PCR (RT-PCR) using a PrimeScriptII First Strand cDNA Synthesis Kit (Takara) with random hexamers after gDNA was removed using a PrimeScript RT reagent Kit with gDNA Eraser. The abundances of bacterial (Primers P1/P2 [28], Table S1) and archaeal 16S rRNA genes (P3/P4 [29]), arsenite S-adenosylmethionine methyltransferase gene (*arsM*) (P9/P10 [30]) in soils, dissimilatory sulfite reductase gene (*dsr*) (P5/P6 [31]), and methyl-coenzyme reductase M (*mcrA*) (P7/P8 [32]) both in soils and enrichment cultures were quantified by quantitative real-time PCR on a CFX96 Thermocycler (Bio-Rad, USA) using DNA and cDNA as template, respectively. The active bacterial and archaeal community structure was analyzed by Illumina High-throughput sequencing using cDNA as template (Supplementary Methods). The sequences were analyzed as described in Chen et al. [33].

### Enrichment cultures of sulfate-reducing bacteria (SRB) and methanogens

The enrichment cultures were established in 100 mL serum bottles containing 40 mL media for SRB or methanogens (Table S2). The media were boiled and flushed with N_2_ and Resazurin (0.01%) was added as a redox indicator dye. For SRB enrichment, acetate (20 mM) or lactate (20 mM) was selected as the electron donor and carbon source, and sulfate (20 mM) was used as the final electron acceptor. Soil (2 g) was collected from the control treatment after flooded incubation for 2 weeks, and transferred into each serum bottle containing SRB media in an anaerobic chamber (95% N_2_ and 5% H_2_). The headspace of the bottles was filled with 20% CO_2_ and 80% N_2_. After three rounds of inoculation, the enrichment cultures were used to test the arsenic methylation ability with the additions of arsenite (5 μM) with or without molybdate (20 mM) or MFP (50 mM) (three replicates per treatment). The bottles were cultured for 1 week at 30 °C without shaking. Arsenic speciation was determined using HPLC-ICP-MS. Sulfide concentration was detected using a methylene blue colorimetry method. For the enrichment cultures of methanogens, acetate, methanol, or methylamine were used as the substrates (5 mM). The enrichment cultures were amended with DMAs (5 μM) with or without BES (5 mM). After incubation for a month at 30 °C without shaking, liquid samples were collected for analysis of As species. Methane in the headspace was collected using a syringe and quantified by gas chromatography. Total DNA was extracted from the SRB and methanogen enrichment cultures, *dsr* and *mcrA* genes were quantified and the composition of *Bacteria* and *Archaea* in the SRB and methanogen enrichment cultures was detected, respectively, as described above.

### Effect of molybdate on ArsM activity in vivo and in vitro

Two aerobic, non-SRB strains of bacteria, *Bacillus* sp. CX-1 and *Pseudomonas alcaligenes* NBRC14159, were used to test whether molybdate inhibits arsenic methylation in vivo. Cells were cultured in LB medium to an OD600 value of 1.0, and transferred to 100 mL fresh LB medium containing 10 μM arsenite with or without 20 mM molybdate. After 24 h, the medium was collected, centrifuged and filtered. Arsenic species in the solution were determined using HPLC-ICP-MS. To test whether molybdate inhibits ArsM activity in vitro, the *arsM* genes (*BlarsM*, *DmarsM*, and *DgarsM*) were amplified from *Bacillus* sp. CX-1 [10], *Desulfosporosinus meridiei* DSM 13257, and *Desulfotomaculum gibsoniae* DSM 7213 using the primers P11-P16 (Table S1). The PCR products were cloned into the vector pET29a(+) and transformed into the *E. coli* strain BL21 (DE3) [10]. ArsMs were extracted and purified following the procedure described in Huang et al. [34]. The arsenite methylation activities of the three ArsMs were assayed in vitro with an equal amount of protein (1.5 μM) [34], with or without the addition of 20 mM molybdate.

### Statistical analysis

The significance of treatment effects was tested by analysis of variance (ANOVA), followed by comparisons of means using Tukey’s test (*P* < 0.05). Where necessary, data were transformed logarithmically prior to ANOVA to stabilize the variance.

## Results

### Soil properties and rice As speciation

CZ and QY soils contained high levels of total As due to mining (CZ) or geogenic mineralization (QY), whereas XY soil was uncontaminated with a very low level of total As (Table 1). XY soil also had a lower pH and a lower SOM than CZ and QY soils. Rice grown in XY paddy field showed severe symptoms of straighthead disease, with unfilled grains and parrot-beak shaped husks (Fig. S1). No such symptoms were observed in CZ and QY. Analysis of As speciation in rice husks showed predominance of DMAs in the XY samples (78%), compared with < 10% DMAs in CZ and QY samples which contained mainly inorganic As (Table 1).

**Table 1:** Selected properties of paddy soils used in the study and arsenic species in the husks of rice collected from three paddy fields

Soil	Total As (mg kg^−1^)	Soil texture	Soil pH	Extractable SO_4_^2−^ (mg kg^−1^)	Soil organic matter (g kg^−1^)	Arsenic species in rice husks			Rice straighthead symptoms in the field
						DMAs (mg kg^−1^)	iAs (mg kg^−1^)	DMAs as % of total As	
CZ	86	Clay	6.7	432	30.8	0.17 ± 0.01	2.07 ± 0.17	7.8	No
QY	101	Clay	6.0	374	21.9	0.19 ± 0.02	2.88 ± 0.13	6.3	No
XY	3.4	Loam clay	5.4	238	11.1	1.50 ± 0.08	0.43 ± 0.12	77.7	Yes

### Microbial functional groups involved in arsenic methylation and demethylation in paddy soils

**Fig. 1:**
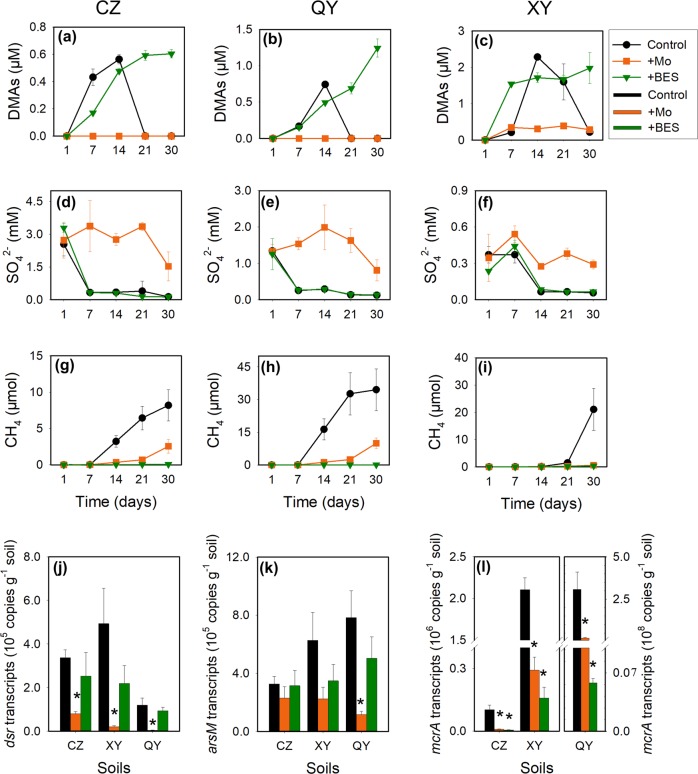
Arsenic methylation and demethylation in three paddy soils. The effects of molybdate and BES on the dynamics of DMAs (**a**–**c**), SO_4_^2−^ (**d**–**f**) in porewater, CH_4_ (**g**–**i**) production from soils and the abundance of *dsr* (**j**), *arsM* (**k**), *mcrA* (**l**) transcripts of CZ (**a**, **d**, **g**, **j**), QY (**b**, **e**, **h**, **k**), and XY (**c**, **f**, **i**, **l**) paddy soils. Transcript levels of functional genes were quantified on day 14 after incubation. Data are means ± SE (*n* = 3). * in (**j**, **k**, **l**) denotes significant difference at *P* < 0.05 (Tukey’s test)

Because microbial As methylation in paddy soils is enhanced under flooded conditions [3], we hypothesized that either SRB or methanogenic archaea were responsible for As methylation. To test this hypothesis, we first determined the dynamics of methylated As species in the porewater of the three paddy soils incubated under flooded conditions with or without the addition of the inhibitors of SRB (Mo) or methanogens (BES). As expected, flooding of soils resulted in decreases in the redox potential and nitrate concentrations, and increases in Fe and iAs (mainly arsenite) concentrations in the porewater (Fig. S2). These changes were similar to those observed in flooded soils with rice plants growing [35, 36], suggesting that the incubation method can mimic the paddy soil conditions. In the control without inhibitors, DMAs was produced in all three soils, reaching a peak at 2 weeks after incubation, which then disappeared during the subsequent 1–2 weeks of incubation (Fig. 1a–c). At the peak of DMAs production, porewater DMAs concentration ranged from 9% (CZ and QY) to 330% (XY) of the iAs concentration. No other methylated As species were detected. Despite much lower concentrations of total soil As and porewater iAs, XY soil produced three to four times higher DMAs concentration than the other two soils. DMAs also disappeared more slowly in XY soil than the other two soils. These results were consistent with the As speciation data in rice husks from the three sites (Table 1).

The addition of molybdate inhibited the production of DMAs completely in CZ and QY soils and by 86.5% at the peak in XY soil (Fig. 1a–c). Molybdate also inhibited the disappearance of sulfate (Fig. 1d–f), consistent with its effect as a SRB inhibitor. In contrast, the primary effect of BES addition was to arrest the disappearance of DMAs after 2 weeks of incubation (Fig. 1a–c). The inhibition of methane production by BES was complete in all three soils, whilst molybdate addition also delayed the production of methane (Fig. 1g–i). A slower production of methane in XY soil can be explained by a slower decrease of the redox potential in this soil (Fig. S2), which also coincided with a slower disappearance of DMAs (Fig. 1c). These results suggest that SRB and methanogens were involved in As methylation and the disappearance of DMAs, respectively.

To further associate SRB and methanogens with As methylation and demethylation, we determined the activities of SRB and methanogens in the three paddy soils. Total RNA was extracted from the soils after flooded incubation for 2 weeks, and the transcript abundances of *dsr* and *mcrA*, the genetic markers of SRB and methanogens, respectively, as well as *arsM*, were quantified by quantitative RT-PCR. The additions of molybdate and BES greatly decreased the transcript abundance of *dsr* and *mcrA*, respectively (Fig. 1j, l), indicating that the active SRB and methanogens had been suppressed by the two respective inhibitors. The results support the interpretation that suppression of SRB and methanogens led to an inhibition of As methylation and disappearance of DMAs, respectively. Molybdate also suppressed *mcrA* transcript and methane production to some extent, but methanogens were not the major driver of As methylation in the three soils because an almost complete inhibition of methanogenic activities by BES did not suppress As methylation. The addition of Mo, but not BES, also resulted in a decrease (29.5–95.2%) in the transcript abundance of *arsM* in all three soils, with the effect being significant (*P* < 0.05) in QY soil (Fig. 1k).

**Fig. 2:**
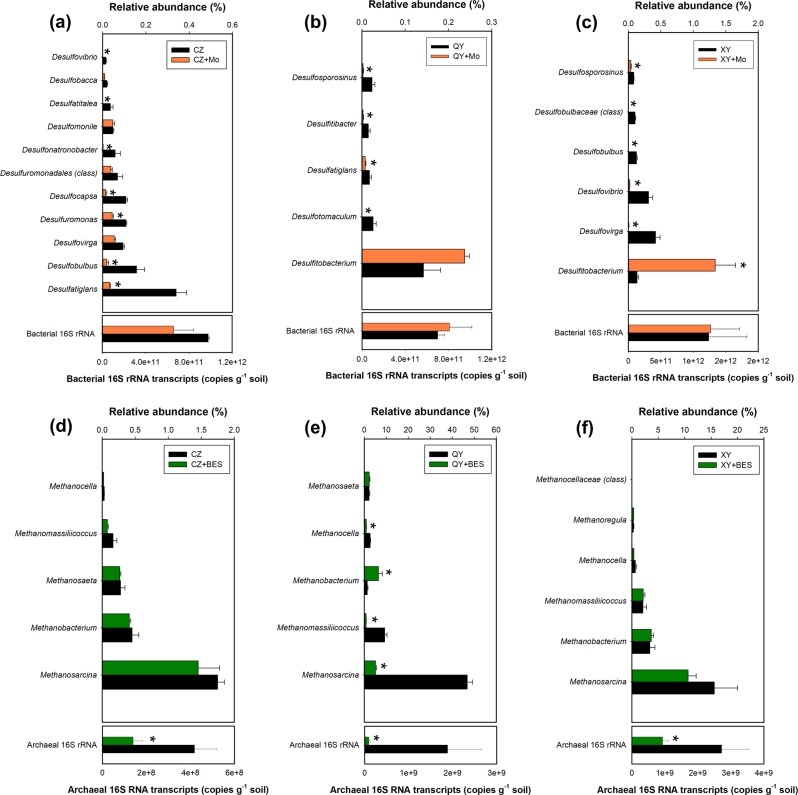
Effects of Mo and BES additions on the abundance of bacterial and archaeal 16S rRNA transcripts and the relative abundance of the active core genera of sulfate-reducing bacteria (**a**–**c**) and methanogens (**d**–**f**) in three paddy soils after incubation for 14 days. Data are means ± SE (*n* = 3). * denotes significant difference at *P* < 0.05 (Tukey’s test)

To find the affiliated phyla of SRB and methanogens that function in As methylation and demethylation, we sequenced the bacterial and archaeal 16S rRNA transcripts in the three soils after incubation for 2 weeks. Sixteen genera of SRB in total were identified in the three soils, among which 14 genera were suppressed by molybdate (Fig. 2a–c). Among the three soils, XY soil had the highest transcript level of *dsr* (Fig. 1j) and the highest relative abundances of *Desulfovirga*, *Desulfovibrio*, and *Desulfosporosinus* (Fig. 2a–c). Seven genera of methanogens were identified in the three soils, among which four genera occurred in all three soils (Fig. 2d–f). The addition of BES decreased significantly (*P* < 0.05) the total transcript abundance of archaeal 16S rRNAs (by 66–95%), as well as the relative abundance of three genera of methanogens (*Methanosarcina*, *Methanomassiliicoccus*, and *Methanocella*) in QY soil, but had relatively little effect on the relative abundance of other genera of methanogens (Fig. 2d–f).

### The enrichment cultures of SRB from paddy soils can methylate arsenite

**Fig. 3:**
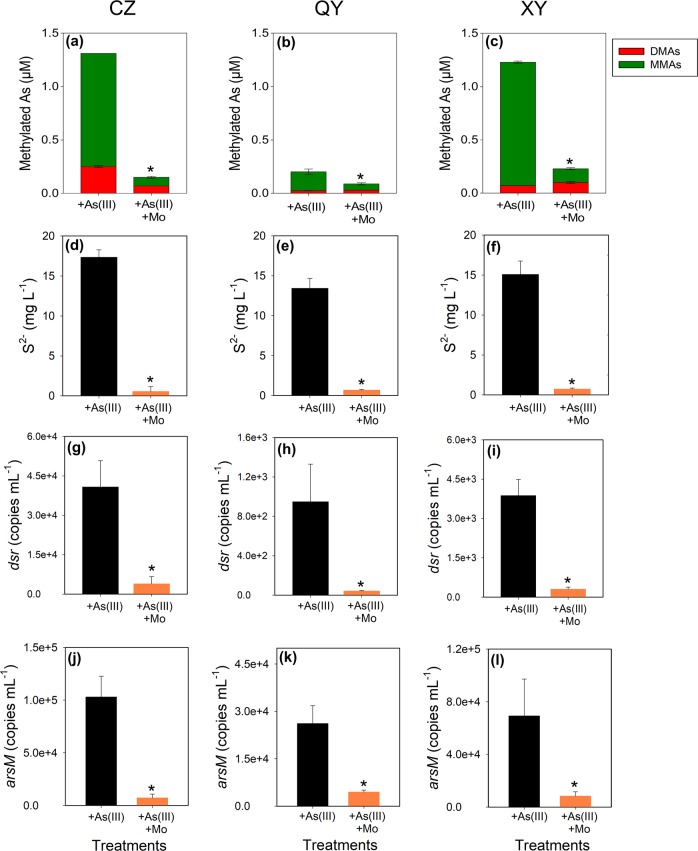
Enrichment cultures of sulfate-reducing bacteria are able to methylate arsenite (5 μM). The effect of molybdate on As methylation (**a**–**c**), sulfide production (**d**–**f**), the abundance of *dsr* gene (**g**–**i**), and *arsM* gene (**j**–**l**) in the enrichment cultures of sulfate-reducing bacteria from CZ (**a**, **d**, **g**, **j**), QY (**b**, **e**, **h**, **k**), and XY (**c**, **f**, **i**, **l**) paddy soils after incubation for 1 week. Data are means ± SE (*n* = 3). * denotes significant difference at *P* < 0.05 (Tukey’s test)

To further investigate whether SRB can methylate As, we established enrichment cultures of SRB containing some key genera of SRB (Table S3) from the three soils using acetate or lactate as the electron donor, and sulfate as the electron acceptor. After 7-day culture, the enrichments from the three soils were all able to methylate arsenite into MMAs and DMAs, with the former being the main product (Fig. 3a–c for the cultures with acetate, and Fig. S3 for the cultures with lactate). The addition of molybdate not only greatly (*P* < 0.05) suppressed the copy number of the *dsr* and *arsM* genes, but also decreased sulfate reduction to sulfide and the methylation of arsenite (Fig. 3). The lower As methylation in the SRB enrichment from QY soil was consistent with its lower copy number of the *dsr* gene and lower abundance of SRB.

Although molybdate is generally considered to be an inhibitor of SRB with a high degree of specificity [37], there is a possibility that it may affect biochemical processes other than sulfate reduction and, consequently, the inhibition of As methylation by molybdate might be unrelated to SRB [30]. To address this question, we first tested a different SRB inhibitor, MFP (monofluorophosphate) [25, 30]. A preliminary experiment showed that a high concentration (500 mmol kg^−1^) of MFP was needed to inhibit sulfate reduction in the soils, possibly because the phosphate-containing MFP was strongly adsorbed by the soil solid phase. The addition of MFP (500 mmol kg^−1^) inhibited sulfate reduction and DMAs production in all three soils (Fig. S4), although the inhibition was not complete as in the case of molybdate addition. We also tested the effect of MFP addition on As(III) methylation in the SRB enrichment cultures from the three soils. An addition of 50 mM MFP decreased the production of sulfide and As methylation by 32–63% and 27–41%, respectively (Fig. S5). MFP also decreased the copy numbers of *dsr* and *arsM* (Fig. S5). These results indicate that MFP can inhibit sulfate reduction and arsenite methylation in both soils and the SRB enrichment cultures, though not as effectively as molybdate. Our results are different from those of Reid et al. [30], who found that MFP inhibited SRB activity but not As methylation in an enrichment culture from a paddy soil. Second, we tested the effect of molybdate on ArsM activities using both in vitro and in vivo assays, as Reid et al. [30] suggested that molybdate might inhibit As methylation by ArsM proteins directly. Three *arsM* genes were cloned from *Bacillus* sp. CX-1 [34] and two SRB, *Desulfosporosinus meridiei* DSM 13257 and *Desulfotonmaculum gibsoniae* DSM 7213 into *Escherichia coli* BL21 (Fig. S6). The three purified ArsM proteins from *E. coli* were tested for arsenite methylation activities in the presence or absence of 20 mM molybdate. It was found that molybdate had no significant effect on the *D. gibsoniae* ArsM activity, but had a relatively small effect on the activities of ArsMs from *Bacillus* sp. and *D. meridiei* (7.3% and 21.8% inhibition, respectively) (Fig. S6). This effect could not explain the complete inhibition of As methylation by molybdate in the soil incubation experiment (Fig. 1). Different from the SRB enrichment cultures, DMAs was the main product of As methylation in the in vitro assays (Fig. S6). Similar results have been reported with a SRB strain (*Clostridium* sp BXM) that produced MMAs in vivo but DMAs in vitro with the purified ArsM protein [38], possibly because abundant amounts of substrates were added to the in vitro assay. It is possible that molybdate treatment may deplete co-substrates (e.g., SAM) required for ArsM activity in vivo. To address this question, we tested the effect of molybdate on arsenite methylation by two strains of aerobic, non-SRB bacteria (*Bacillus* sp. CX-1 and *Pseudomonas alcaligenes* NBRC14159). Molybdate (20 mM) had little effect on arsenite methylation by these strains of bacteria in vivo (Fig. S7). Taken together, the results indicate that molybdate inhibits As methylation in paddy soils mainly through its effect on SRB.

### Exogenous DMAs is demethylated by methanogens in paddy soils

**Fig. 4:**
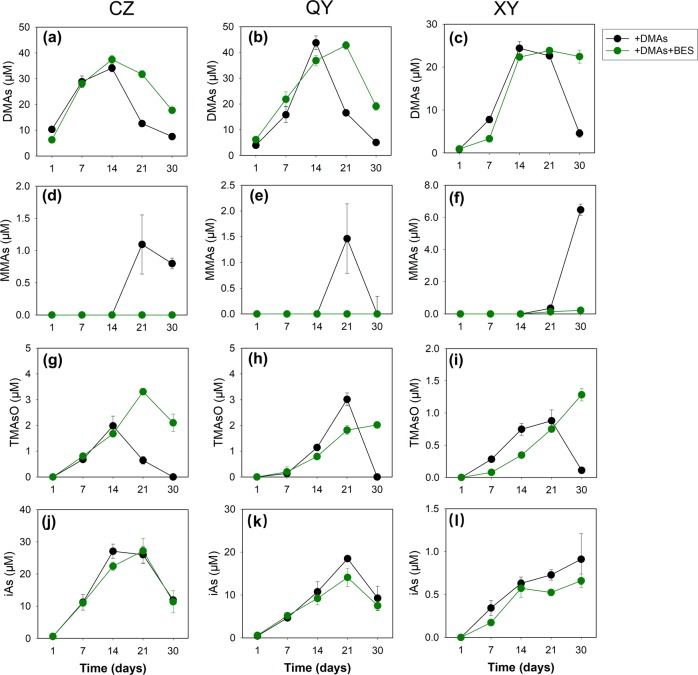
Demethylation of exogenous DMAs (40 μmol kg^−1^). The effect of BES on the disappearance of exogenous DMAs (**a**–**c**) and the dynamics of MMAs (**d**–**f**), TMAsO (**g**–**i**), and iAs (**j**–**l**) in the porewater from CZ (**a**, **d**, **g**, **j**), QY (**b**, **e**, **h**, **k**), and XY (**c**, **f**, **i**, **l**) paddy soils. Data are means ± SE (n = 3)

The soil incubation experiments described above were conducted without the additions of exogenous methylated As compounds. To test whether exogenous DMAs added to paddy soils could also be demethylated by methanogens, we conducted a soil incubation experiment with an addition of 40 μmol kg^−1^ DMAs in the presence or absence (control) of BES. In both treatments, porewater DMAs concentration increased during the first two weeks of incubation to 25–40 μM, accounting for 63–100% of the DMAs added (Fig. 4a–c). These results suggest that most of the exogenous DMAs was initially adsorbed on the soil solid phase and then released into the porewater during flooded incubation, likely as a result of the reductive dissolution of iron oxyhydroxides. DMAs concentration then decreased rapidly in the control, and concurrently MMAs was produced (Fig. 4d–f), suggesting that some of DMAs had been demethylated to MMAs. MMAs concentration showed a decrease in CZ and QY soils after 3 weeks, suggesting that it was further demethylated. The addition of BES slowed the disappearance of the exogenous DMAs and almost completely blocked MMAs production. Exogenous DMAs may also undergo further methylation to TMAsO by some soil microbes [33]. TMAsO was detected in the porewater in all three soils during the first 2–3 weeks of incubation at concentrations equivalent to 3.6–6.9% of the porewater DMAs (Fig. 4g–i). Thereafter, TMAsO disappeared rapidly, but the disappearance of TMAsO was also delayed by the addition of BES. These results can be explained by demethylation of different methylated As species by methanogens. In a further experiment, we determined As species in the soil solution and the solid phase (by extraction with 0.5 M phosphoric acid), and volatile As in the headspace (by chemotrapping) [3], on day 1 and 30 after the addition of exogenous DMAs (Table S4). Volatile As species were not detectable on either day 1 or day 30. During the 30-day incubation, most of the added DMAs had been demethylated to MMAs and iAs; the latter was mainly sorbed on the solid phase. There was a reasonable mass balance of As species between day 1 and day 30 in all three soils.

### Methanogenic enrichments of paddy soils can demethylate DMAs

**Fig. 5:**
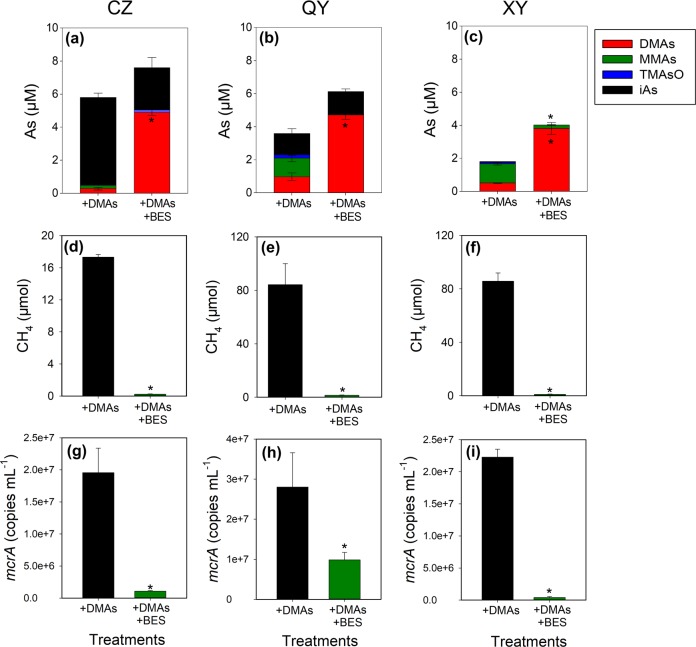
Enrichment cultures of methanogens are able to demethylate DMAs (5 μM). The effect of BES on the demethylation of DMAs (**a**–**c**), methane production (**d**–**f**), and the abundance of *mcrA* gene (**g**–**i**) in the enrichment cultures of methanogens from CZ (**a**, **d**, **g**), QY (**b**, **e**, **h**), and XY (**c**, **f**, **i**) paddy soils after incubation for 30 days. Data are means ± SE (*n* = 3). * denotes significant difference at *P* < 0.05 (Tukey’s test)

To gain a better understanding of DMAs demethylation, we established enrichment cultures of methanogens with 5 mM of acetate as the substrate from the three paddy soils. The culture contained four core genera of methanogens with a total relative abundance of 10.8–73.5% (Table S5). After 30 days, all three enrichments demethylated 80–90% of the added DMAs (5 μM) to MMAs and iAs. The addition of BES to the enrichment cultures not only decreased the copy number of *mcrA* (Fig. 5g–i) and inhibited methane production (Fig. 5d–f), but also suppressed DMAs demethylation (Fig. 5a–c). The mass balance of As species in the solution phase of the enrichment culture was below the expected level (5 μM) in some treatments, possibly because of the adsorption of As species, especially the end product iAs, by some soil particles that were still present in the enrichment cultures. Another possibility was the volatilization of methylated As species, but this was ruled out as the amounts of volatile As in the headspace of the enrichment cultures were negligible. It is known that some genera of methanogens can use C1-compounds such as methylamine and methanol as the substrate. These two compounds were also used as the substrate to enrich methanogens. In both cases, DMAs was demethylated in the methanogen enrichment cultures, with the demethylation rate being faster in the culture with methanol as the substrate (Fig. S8). In a further experiment with the addition of MMAs, the methanogen enrichment culture from CZ soil was able to demethylate MMAs to iAs (Fig. S9).

### ^13^C-labeled DMAs is demethylated to produce ^13^C-methane

**Fig. 6:**
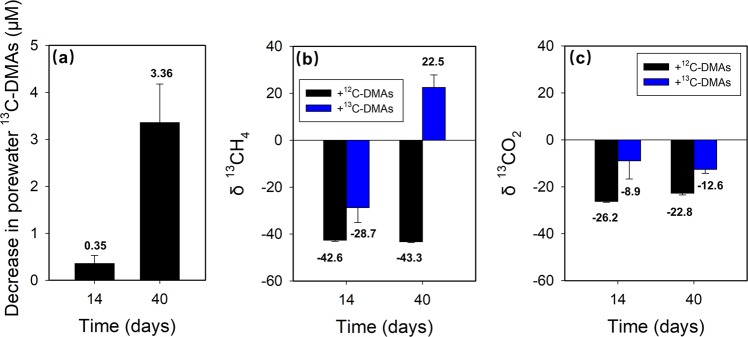
Demethylation of ^13^C-labeled DMAs. Decrease in the concentration of ^13^C-DMAs in soil porewater (**a**) and changes in the δ^13^CH_4_ (**b**) and δ^13^CO_2_ (**c**) in the headspace after incubation of CZ soil for 14 and 40 days. Data are means ± SE (*n* = 3)

To provide direct evidence that DMAs is demethylated by methanogens, we synthesized ^13^C-labeled DMAs (Supplementary Methods and Fig. S10). ^13^C-DMAs was then added to CZ soil incubated under flooded conditions with the addition of non-labeled DMAs serving as the control. After 14 and 40 days of incubation, the concentration of ^13^C-DMAs in soil porewater decreased from the initial level of 5 μM, whilst ^13^C-methane was produced in the headspace (Fig. 6a, b). In the non-labeled DMAs treatment, δ^13^C of the methane produced was −42.62 ± 0.84‰ and −43.29 ± 0.58‰ on day 14 and 40, respectively (Fig. 6b). With the addition of ^13^C-DMAs, δ^13^C of the methane increased to −28.74 ± 11.07‰ and 22.52 ± 9.18‰ on day 14 and 40, respectively. There was a reasonable agreement between the amount of ^13^C-methane produced and the disappearance of ^13^C-DMAs from the porewater (95 ± 11%, assuming that demethylation of four methyl groups would generate three molecules of methane; see Discussion). In addition, we detected an increase of δ^13^C of CO_2_ in the headspace from the control of −26.21 ± 0.70‰ and −22.75 ± 1.21 to −8.92 ± 13.45‰ and −12.58 ± 2.93‰ in the ^13^C-DMAs treatment on day 14 and 40, respectively (Fig. 6c). Because ^13^CO_2_ concentration was not determined, mass balance calculation could not be done for the conversion of ^13^C-DMAs to ^13^CO_2_. This experiment demonstrated that ^13^C-DMAs had been demethylated to produce ^13^C-labeled methane and ^13^C-labeled carbon dioxide by methanogens.

## Discussion

In flooded incubation of three paddy soils, we found that DMAs accumulated in soil porewater during the initial 2–3 weeks of flooding, followed by its rapid disappearance in the subsequent weeks (Fig. 1). The amount of DMAs produced was not related to the soil total As concentration or the porewater iAs concentration. The XY soil was collected from a paddy field that produced severe straighthead disease in rice, characterized by a high accumulation of DMAs in the panicles (Table 1). This soil, despite a low total As concentration, produced a large amount of DMAs in the porewater (Fig. 1). Our results support the notion that the ability to produce methylated As species, especially DMAs, is an important factor for the induction of rice straighthead disease [16].

Our results showed that some SRB were involved in As methylation in flooded paddy soils. Inhibition of SRB by molybdate and MFP inhibited arsenite methylation in both soils and the SRB enrichment cultures, with the extent of inhibition being consistent with their effects on sulfate reduction (Figs. 1 and 3, Figs. S4 and S5). The SRB enrichment cultures from the three soils showed the ability to methylate iAs (Fig. 3). MMAs, instead of DMAs, was the main methylation product in the SRB enrichment cultures, perhaps because the enrichment cultures captured only a small subset of SRB. Another possible explanation is that SRB may mainly mediate the first step of arsenite methylation to MMAs, which is then used by other microbes to produce DMAs. In support of this explanation, a soil SRB (*Clostridium* sp. BXM) was found to produce mainly MMAs in pure culture [38], whilst microbial ArsMs that can efficiently methylate MMAs, but not arsenite, have also been reported [34, 39]. There is also a possibility that the presence of some microbes may enhance arsenite methylation by SRB, such as an enhanced mercury methylation by SRB in the presence of *Syntrophobacter* [40]. The finding that SRB play a role in arsenite methylation in flooded paddy soils does not exclude the contribution from other microbial groups, especially the possibility of other microbes in methylating MMAs to DMAs.

We showed that DMAs produced in paddy soils or added exogenously was demethylated by methanogenic archaea, concurrent with the production of methane. Inhibition of methanogens by BES inhibited the demethylation of DMAs, as well as other methylarsenic species such as MMAs and TMAsO (Figs. 1 and 4). The enrichment cultures of methanogens from all three soils were able to demethylate DMAs (Fig. 5, Fig. S8). Importantly, ^13^C-labeled DMAs was demethylated to produce ^13^C-labeled methane (Fig. 6), providing direct evidence for the involvement of methanogens in DMAs demethylation. It is known that some methylotrophic methanogens in the order Methanosarcinales are able to use methyl-group containing compounds (e.g., methanol, methylated amines, methylated sulfides, and methylated selenides) as the substrate to produce methane [41–44]. In the three paddy soils tested, methylotrophic methanogens, including *Methanosarcina* and *Methanomassiliicoccus*, were detected, accounting for 1.8–46.8% and 0.2–9.2%, respectively, of total archaea (Fig. 2). For the first time, our study has shown that methylated arsenic species can also be demethylated by methylotrophic methanogens. The mechanism of methylotrophic methanogenesis involves the transfer of methyl groups from methylated compounds to a cognate corrinoid protein and then to Coenzyme M; Methyl-CoM subsequently enters the methanogenesis pathway and is reduced to methane, with the stoichiometry of four methyl groups producing three molecules of methane and one molecule of CO_2_ [43]. It is probable that this mechanism also applies to DMAs demethylation, as the amount of ^13^C-methane produced was approximately three-fourth of the ^13^C-DMAs demethylated and ^13^CO_2_ was also detected. This mechanism is different from demethylation of trivalent MMAs catalyzed by C·As lyase in the aerobic bacterium *Bacillus* sp. MD1 [22].

**Fig. 7:**
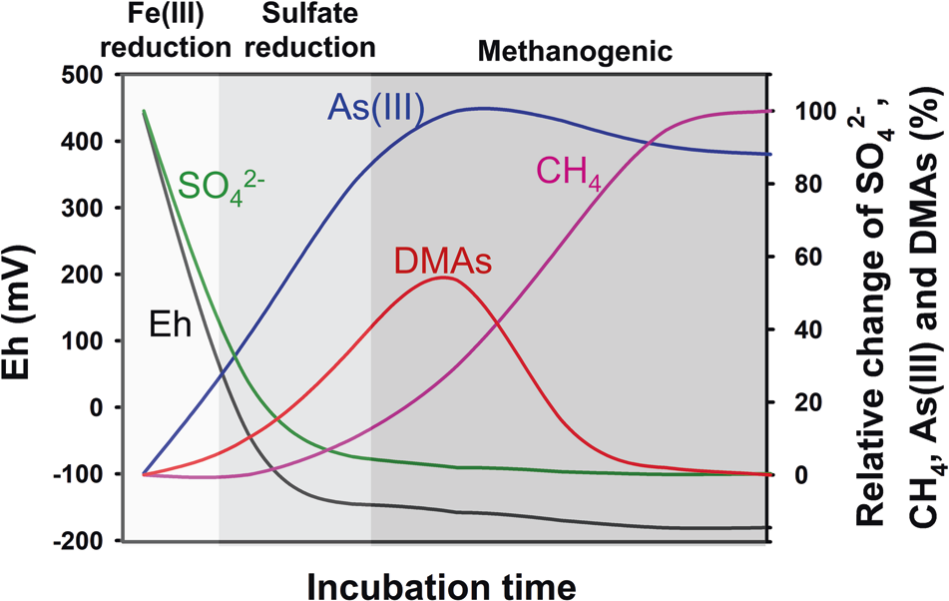
A conceptual biogeochemical model explaining the dynamics of As methylation and demethylation in paddy soil

Based on the experimental results, we present a conceptual biogeochemical model explaining the dynamics of As methylation and demethylation in flooded paddy soil, which explains the roles of SRB and methanogens in these processes due to their ecological niches (Fig. 7). Upon flooding, the decrease in soil redox potential leads to a reductive dissolution of iron oxyhydroxides and a rapid increase in arsenite concentration in the porewater (Fig. S2). Further decrease in the redox potential to the range suitable for SRB coincides with a greatly increased availability of arsenite, the substrate for As methylation. SRB and other anaerobes possessing ArsMs mediate arsenite methylation proceeds rapidly, resulting in an accumulation of DMAs in the porewater. When the redox potential decreases to the range suitable for methanogens, methylotrophic methanogenesis starts, resulting in demethylation of DMAs and other methylated arsenic species. This model explains the dynamic nature of DMAs concentration observed in flooded paddy soils, which is dependent not only on the substrate availability, but also on the balance between the activities of microbes possessing As methylating ability and those of methylotrophic methanogens capable of demethylating DMAs. This model also helps explain why rice straighthead disease can occur in some paddy soils with relatively low concentrations of total As where microbial community composition may favor As methylation and/or disfavor demethylation of DMAs. Based on the insight from the present study, rice straighthead disease can be prevented or alleviated by decreasing the formation of DMAs or promotion of the degradation of DMAs in paddy soil. Mid-season draining of paddy water has been shown to be an effective measure to control straighthead disease [45, 46]. This can be explained by the fact that draining of paddy water raises soil redox potential and decreases the arsenite availability and the activities of SRB and other anaerobes for arsenite methylation. Alternatively, enhancing the activities of methylotrophic methanogens may offer another possible way to control DMAs accumulation in paddy soils and in rice plants. The biogeochemical model presented for As methylation–demethylation cycle in paddy soil is likely to be applicable to other wetland systems, therefore enhancing the understanding of the global As biogeochemical cycle.

## Supplementary information

Supplementary materials
