# Supplementary material for: Sulfate-reducing bacteria and methanogens are involved in arsenic methylation and demethylation in paddy soils
Source: ISME J. 2019 Jun 21;13(10):2523–35. doi: 10.1038/s41396-019-0451-7 (PMC6776024; doi:10.1038/s41396-019-0451-7)
Supplement: Supplementary file 1 — Supplementary materials [file 41396_2019_451_MOESM1_ESM.pdf]

Supplementary Materials

Sulfate-reducing bacteria and methanogens are involved in arsenic methylation and demethylation in paddy soils

Chuan Chen, Lingyan Li, Ke Huang, Jun Zhang, Wan-Ying Xie, Yahai Lu, Xiuzhu Dong and Fang-Jie Zhao

Supplementary Methods

Analysis of soil properties

Total As concentration was determined using inductively-coupled plasma mass spectrometry (ICP-MS, Perkin Elmer NexION 300x, USA) after digestion with aqua regia ($HCl:HNO_3$ 4:1 v/v) [1]. Soil organic matter (SOM), texture and pH were determined using standard methods [2]. Sulfate was extracted using deionized water and determined using ion-chromatography.

Sequencing of bacterial and archaeal 16S rRNA

cDNA synthesized using RNA extracted from the three soils and DNA extracted from the SRB and methanogen enrichment cultures were used to analyze the composition of bacteria and archaea. Bacterial and archaeal 16S rRNA genes were amplified using P1/P2 and P3/P4 (listed in Table S1). Amplicons were purified and quantified. The pooled products were submitted to an Illumina Miseq platform (Shanghai Biozeron). The raw data were quality-filtered, assembled and processed using Quantitative Insights Into Microbial Ecology (QIIME). Operational Units (OTUs) were clustered with 97% similarity cutoff using UPARSE. The phylogenetic affiliation of each 16S rRNA gene sequence was identified by RDP Classifier (http://rdp.cme.msu.edu/) against the silva (SSU128)16S rRNA database using the confidence threshold of 70%. The raw sequence data have been deposited in the NCBI SRA database under the accession numbers SRP151514 (bacteria in soils), SRP151540 (archaea in soils), SRP162388 (bacteria in enrichment cultures of SRB) and SRP162389 (archaea in enrichment cultures of methanogens).

Mass balance of arsenic species in soil incubation after the addition of DMAs

To determine the mass balance and the distribution of As species in the solution, solid and gas phases, an incubation experiment was set up using the three paddy soils amended with 40 μmol kg^{-1} DMAs. Twenty g of soil were mixed with DMAs and placed into a 100 mL serum bottle, to which 40 mL deionized water was added. Each soil was replicated in 3 bottles. The bottles were sealed using rubber stoppers, secured with aluminum crimp caps and incubated at 25 ^{o}C in the darkness. On day 1 and 30, 2 mL mixture of the soil and water suspension were collected into 15 mL tubes and centrifuged. The soil solution was collected, acidified with concentrated HCl to pH below 2.0 and filtered. Arsenic species sorbed in the soil solid phase were extracted using 0.5 M phosphoric acid (1:10) in a shaker (200 rpm) for 12 hours at 25 ^{o}C. After extraction, the mixture was centrifuged. The supernatant was

acidified with HCl and filtered. Arsenic species in the solutions were determined using HPLC-ICP-MS. Volatile As species in the headspace were purged and trapped using chemotraps with silica gels impregnated with 10% (w/v) $AgNO_3$ [3]. Arsenic species trapped on the silica gel were extracted with 1% (v/v) HNO_3 in microwave digestor system (CEM MARS6). The extract was filtered and oxidized using 30% H_2O_2 and arsenic species were detected using HPLC-ICP-MS [3]. The recovery of DMAs added exogenously into three soils ranged from 93.7 – 96.8% by extraction with 0.5 M phosphoric acid according to the method described by Liu et al. [4].

Enzymatic synthesis and purification of ^{13}C-labeled DMAs

^{13}C-labeled DMAs was synthesized via two enzymatic reactions. In the first step, ^{13}C-methyl-SAM was synthesized from ^{13}C-methyl-methionine by SAM synthetase (MetK) according to Dowling et al.[5] The SAM synthetase gene (*metK*) was amplified from *E. coli* strain K12 MG1655 using the primer P17/P18 (Table S1) and cloned into pET29a(+). The plasmid was transformed into the *E. coli* strain BL21(DE3). The transformed strain was inoculated, induced and grown overnight at 16 °C. Cells were harvested, washed, resuspended and lysed. Cleared lysate was loaded on a Ni-NTA column and MetK was eluted by a Tris-HCl (50 mM, pH 7.4) buffer containing 150 mM imidazole. MetK was identified using SDS-PAGE (Fig. S10a) and the concentration of MetK was detected by Nanodrop-2000c. ^{13}C-methyl-SAM was generated in a buffer solution containing 50 mM Tris-HCl buffer (pH 7.4), 1.5 μM MetK, 50 mM KCl, 1 mM EDTA, 20% acetonitrile, 26 mM $MgCl_2$, 13 mM ATP, and 10 mM ^{13}C-methyl-methionine. After reaction for 24 h at 30 °C, the mixture was centrifuged at 12000 *g* for 5 mins at 4 °C. ^{13}C-methyl-SAM in the supernatant was determined using HPLC (PerkinElmer series 200) with a C18 column (EC 250/4.6 NUCLEODUR 100-5 C18ec) eluted with 0.14% trifluoroacetic acid and 0.06% heptafluorobutyric acid. SAM was determined by UV at 254 nm (Fig. S10b). In the second step, ^{13}C-DMAs was synthesized from the reaction mixture of the first enzymatic reaction using arsenite *S*-adenosylmethionine methyltransferase from *Bacillus* sp. CX-1 (BlArsM) [6], which catalyzes arsenite methylation to DMAs only. BlArsM was extracted and purified according to the method described by Huang et al. [6] (Fig. S6a). The enzymatic reaction took place in a test tube containing 10 μM As(III), 8 mM GSH, 1 mM ^{13}C-methyl-SAM and 1.5 μM BlArsM at 30 °C. After reaction for 24 h, the solution was adjusted to pH 3.0 with HCl and passed through a cation-exchange column (SCX Polymer 6393828-01, USA) to remove residual ^{13}C-methyl-SAM and ^{13}C-methyl-methionine. The process was repeated for five times. The purified ^{13}C-DMAsin the eluent was identified by HPLC-ICP-MS (Fig. S10c) and HPLC-ESI-MS/MS (Agilent 1200 series HPLC and a G6410B triple quadrupole mass spectrometer) (Fig. S10d). The HPLC-ESI-MS/MS data showed that both methyl groups of DMAs were labeled with ^{13}C (Fig. S10d). In the eluent from the cation exchange column, no ^{13}C-methyl-SAM or ^{13}C-methyl-methionine was detected (Fig. S10e, f), whereas both compounds were present in the reaction mixture before column separation (Fig. S10e, f). Using this method, approximately 4.5 μg of ^{13}C-DMAs was produced.

References

1. McGrath SP, Cunliffe CH. A simplified method for the extraction of the metals Fe, Zn, Cu, Ni, Cd, Pb, Cr, Co and Mn from soils and sewage sludges. J Sci Food Agric. 1985; 36(9):794-8.
2. Sparks DL (1996) *Methods of Soil Analysis. Part 3: Chemical Methods* (Soil Science Society of America, Madison, Wisconsin).
3. Mestrot A, Uroic MK, Plantevin T, Islam MR, Krupp EM, Feldmann J, et al. Quantitative and

qualitative trapping of arsines deployed to assess loss of volatile arsenic from paddy soil. Environ Sci Technol. 2009; 43(21):8270-5.
4. Liu X, Zhang W, Hu Y, Cheng H. Extraction and detection of organoarsenic feed additives and common arsenic species in environmental matrices by HPLC-ICP-MS. Microchem J. 2013; 108:38-45.
5. Dowling DP, Bruender NA, Young AP, McCarty RM, Bandarian V, Drennan CL. Radical SAM enzyme QueE defines a new minimal core fold and metal-dependent mechanism. Nat Chem Biol. 2014; 10(2):106-12.
6. Huang K, Xu Y, Packianathan C, Gao F, Chen C, Zhang J, et al. Arsenic methylation by a novel ArsM As(III) S-adenosylmethionine methyltransferase that requires only two conserved cysteine residues. Mol Microbiol. 2018; 107(2):265-76.

Supplementary Figures

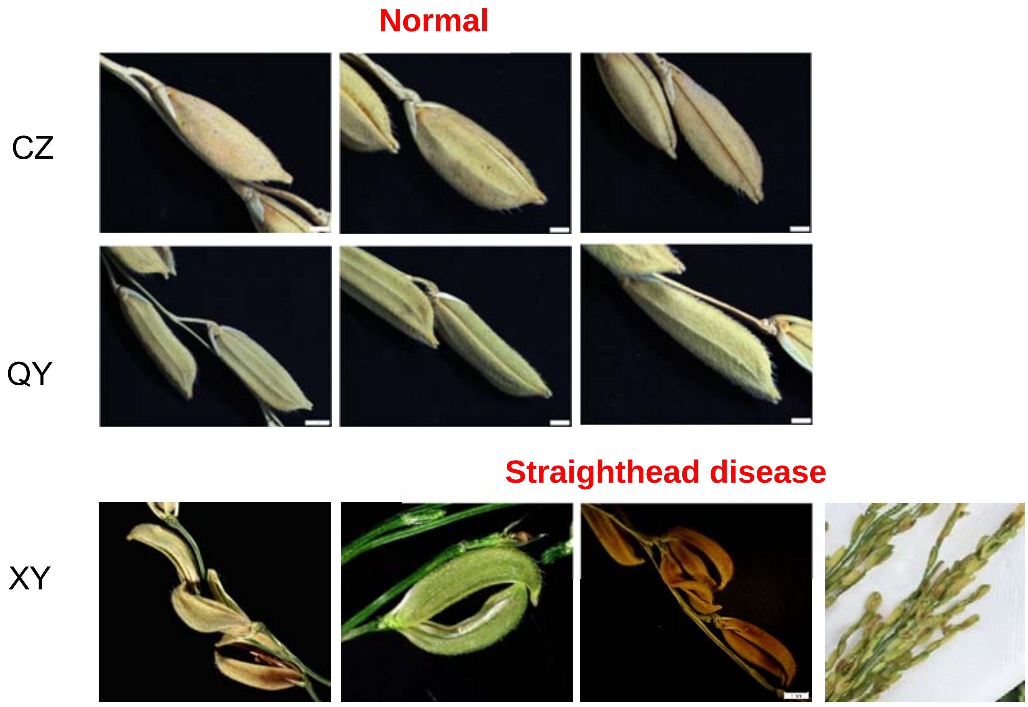

Fig. S1 Symptoms of normal and straighthead disease of rice collected from CZ, QY and XY paddy fields.

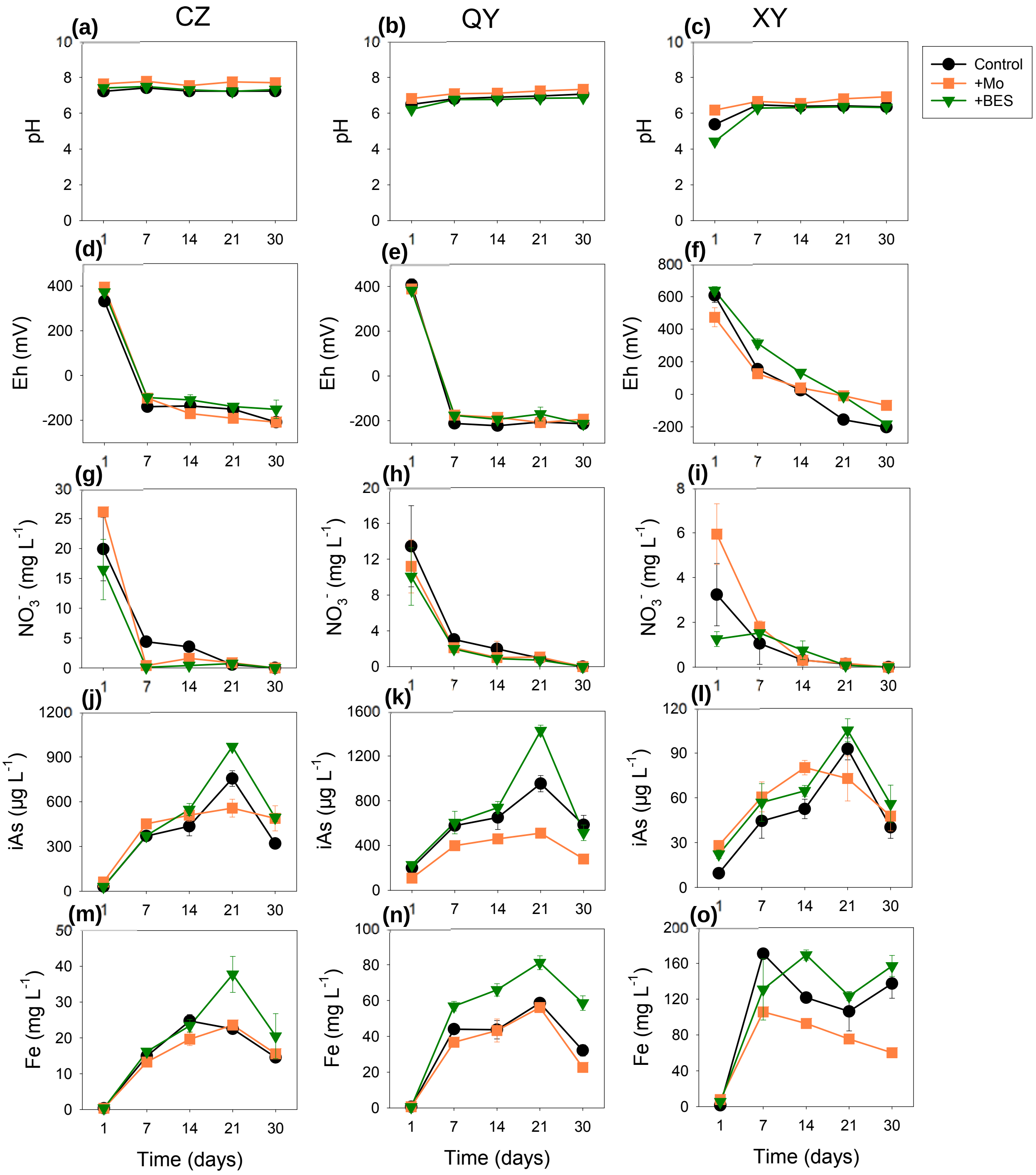

Fig. S2 Effects of molybdate and BES additions on soil pH (**a-c**), Eh (**d-f**), the concentration of nitrate (**g-i**), inorganic As (**j-l**) and total Fe (**m-o**) in the porewaters of CZ (**a**, **d**, **g**, **j**, **m**), QY (**b**, **e**, **h**, **k**, **n**) and XY (**c**, **f**, **i**, **l**, **o**) paddy soils in the incubation experiments. Data are means ± SE (*n* = *3*).

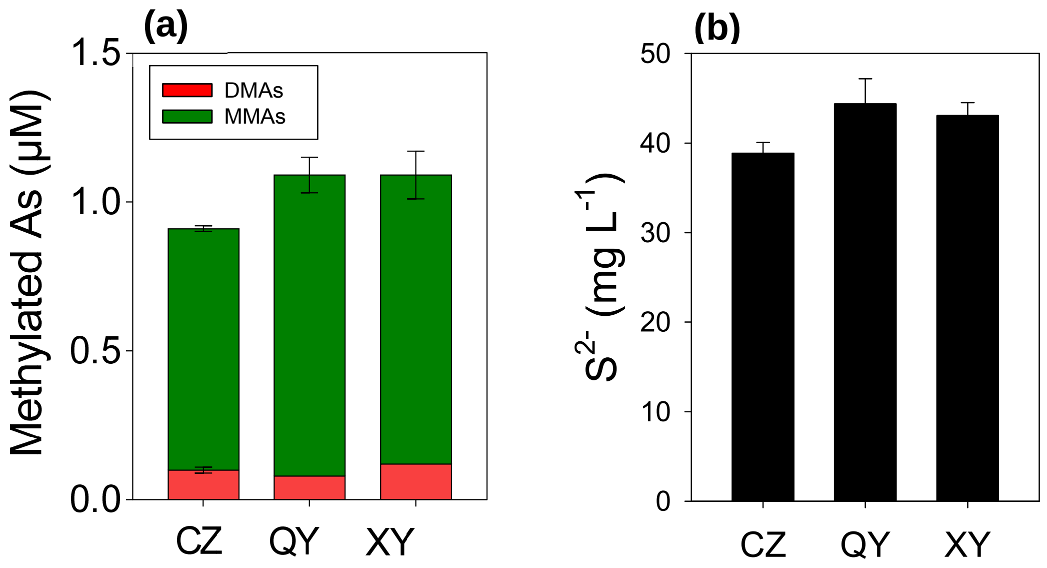

Fig. S3 Arsenite methylation (**a**) and sulfide production (**b**) of the SRB enrichment cultures from the three paddy soils with lactate as the electron donor. Arsenite (5 µM) was added to the enrichemnt cultures and incubated for 7 days before the determination of methylated As species. Data are means ± SE (n = 3).

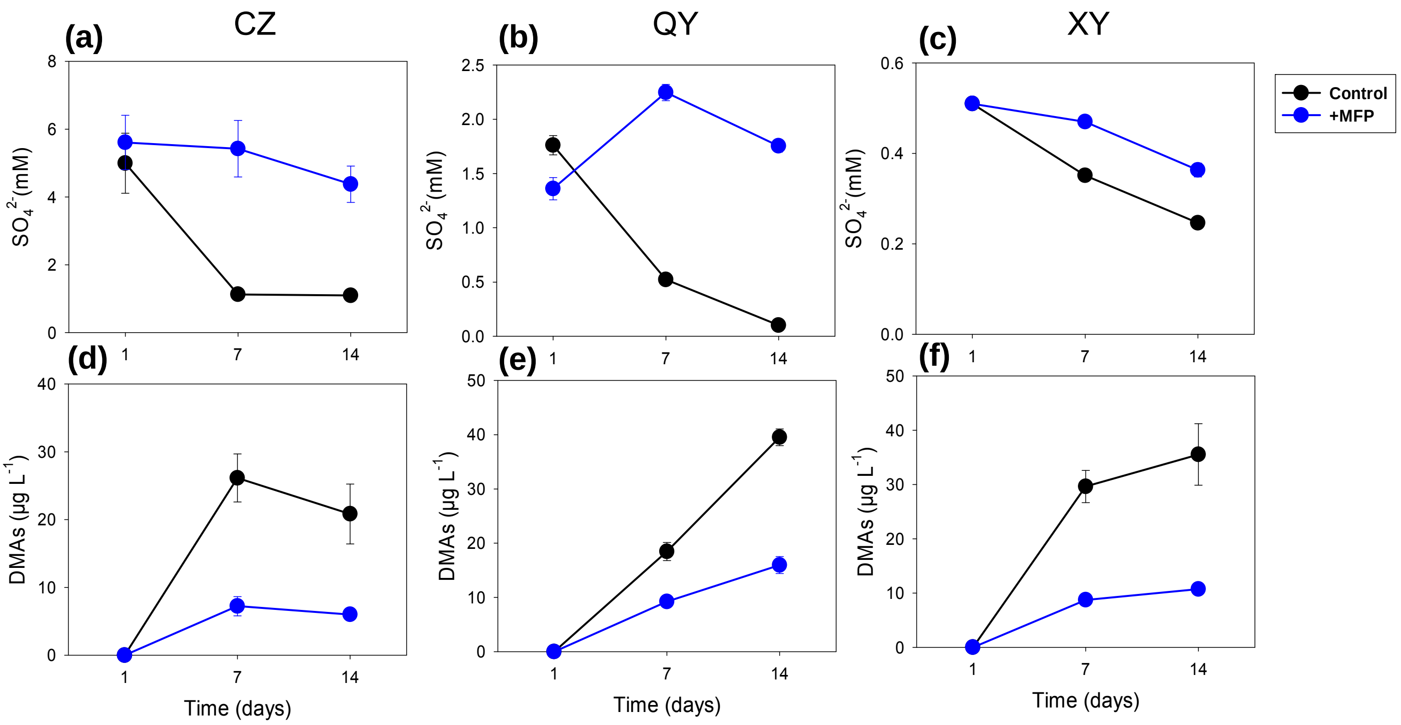

Fig. S4 Effects of monofluorophosphate (MFP, 500 mmol kg^{-1}) on sulfate reduction (**a**-**c**) and arsenic methylation (**d**-**f**) in CZ (**a**, **d**), QY (**b**, **e**) and XY (**c**, **f**) paddy soils. Data are means ± SE (n = 3).

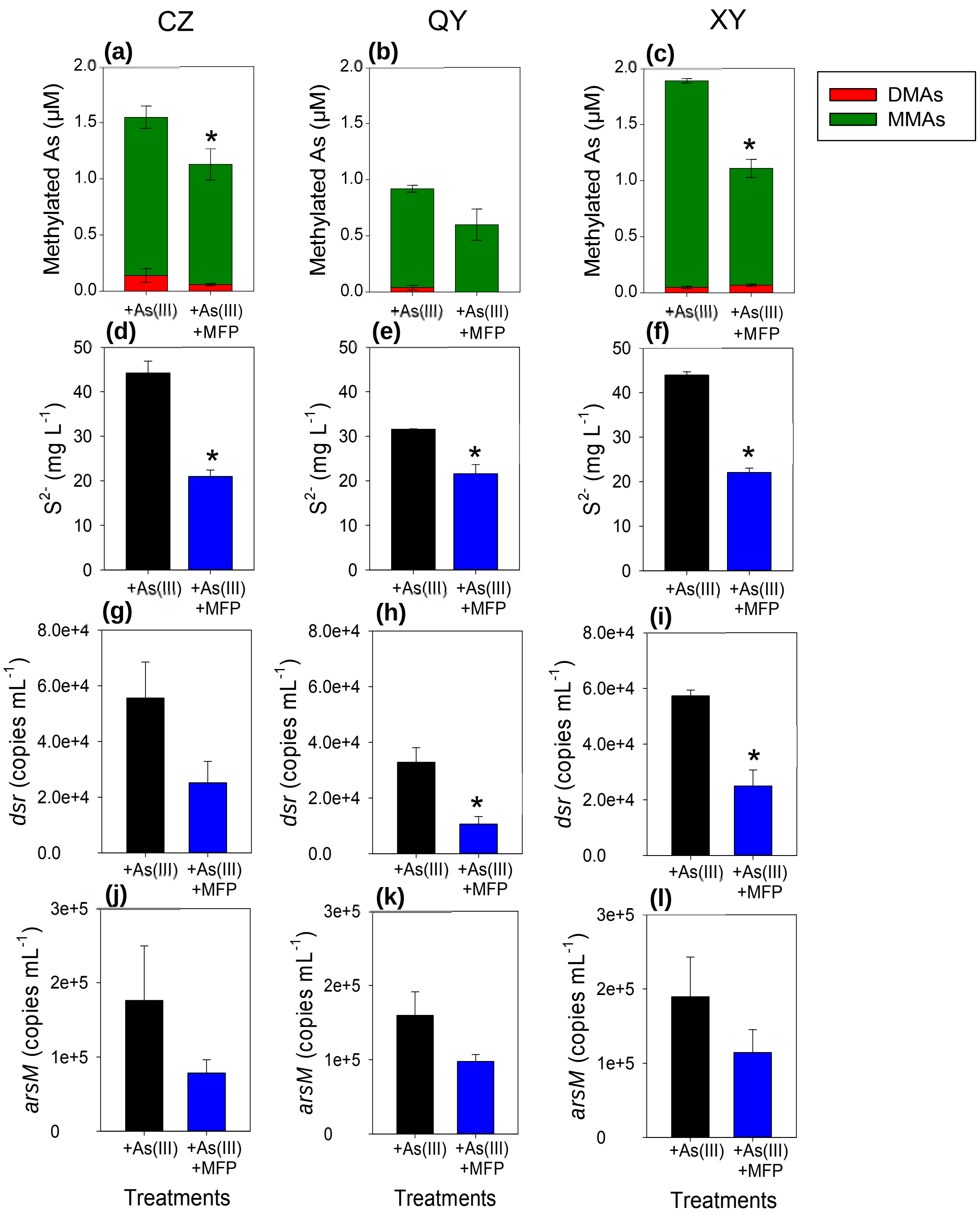

Fig. S5 Effects of monofluorophosphate (MFP, 50 mM) on arsenite methylation (**a**-**c**), sulfide production (**d**-**f**), the abundance of *dsr* gene (**g**-**i**) and *arsM* gene (**j**-**l**) in the SRB enrichment cultures from CZ (**a**, **d**, **g**, **j**), QY (**b**, **e**, **h**, **k**) and XY (**c**, **f**, **i**, **l**) paddy soils. After six rounds of inoculation, the cultures were exposed to 5 µM As(III) and incubated for 7 days. Data are means ± SE (n = 3).

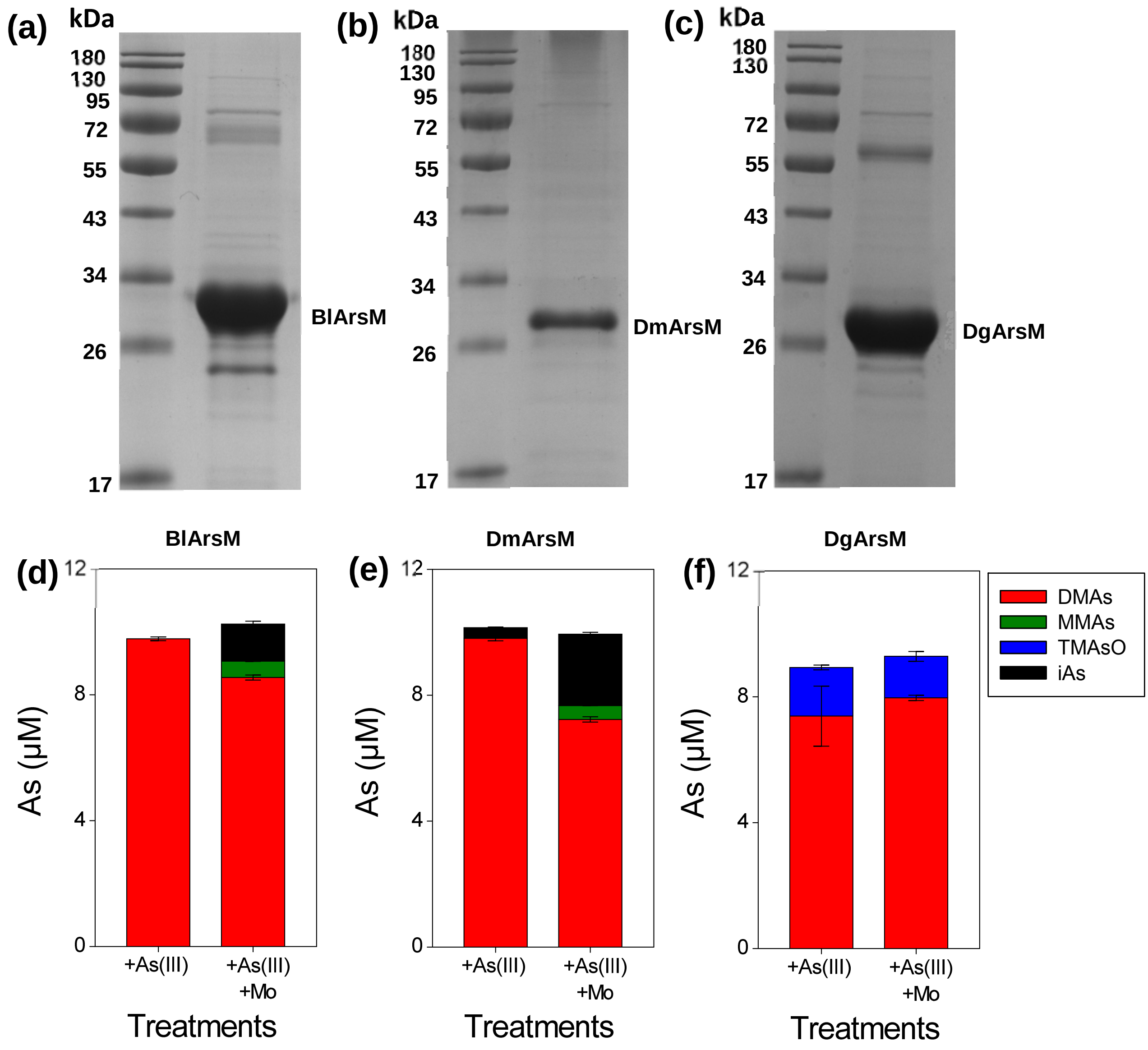

Fig. S6 Effects of molybdate on the arsenite (10 μM) methylation activities of ArsMs in vitro. Three *arsM* genes were cloned from *Bacillus* sp. CX-1 (**a**, **d**), *Desulfosporosinus meridiei* DSM 13257 (**b**, **e**) and *Desulfotomaculum gibsoniae* DSM 7213 (**c**, **f**) and expressed in *E. coli*. ArsMs were purified and identified by SDS-PAGE (**a**-**c**). The arsenite methylation activities of the three ArsMs were determined in vitro with or without the addition of 20 mM Mo (**d**-**f**). Data are means ± SE (*n* = 3).

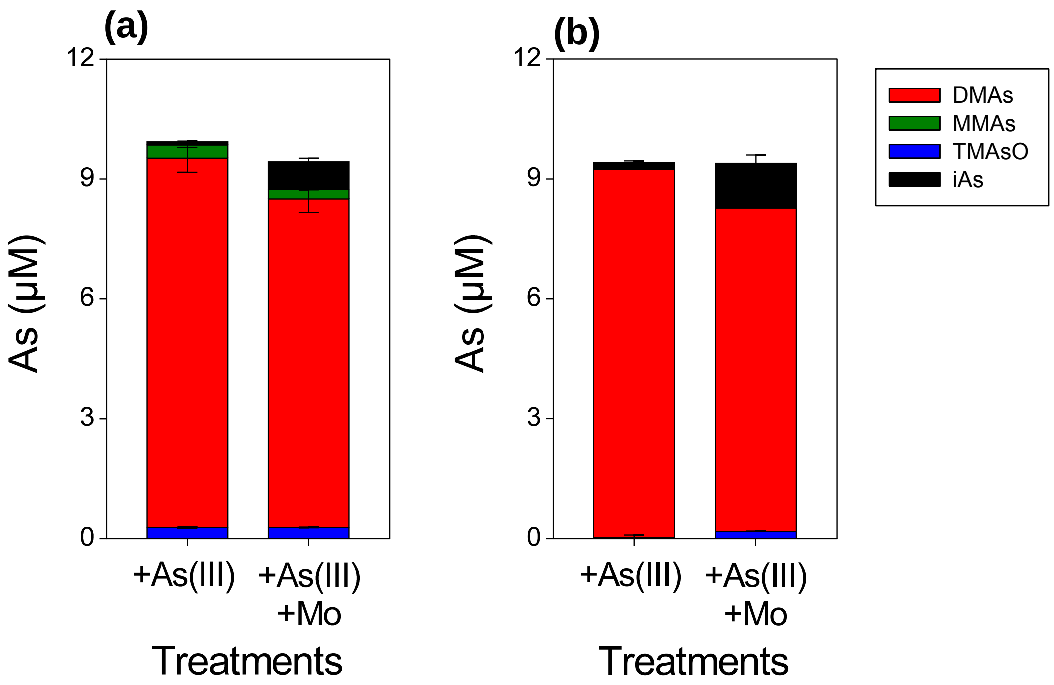

Fig. S7 The effect of molybdate on arsenite methylation in vivo by *Bacillus* sp. CX-1 and *Pseudomonas alcaligenes* NBRC14159. Arsenic species in the cultures of *Bacillus* sp. CX-1 (**a**) and *Pseudomonas alcaligenes* NBRC14159 (**b**) after 24 h culture with 10 μM arsenite with or without 20 mM molybdate. Data are means ± SE ($n = 3$).

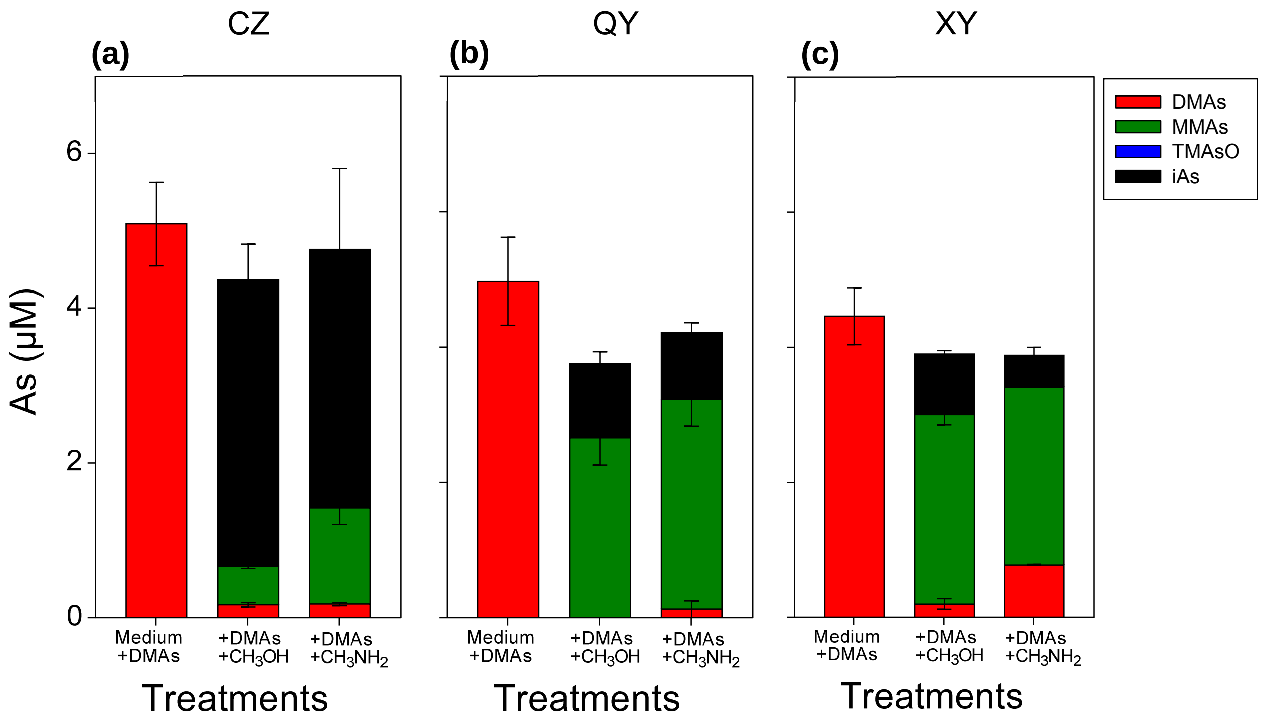

Fig. S8 Demethylation of DMAs in the enrichment cultures of methanogens from CZ (**a**), QY (**b**) and XY(**c**) paddy soils with methylamine or menthol as the substrate. Enrichment cultures were not added to the Medium + DMAs treatment. Data are means ± SE (*n* = *3*).

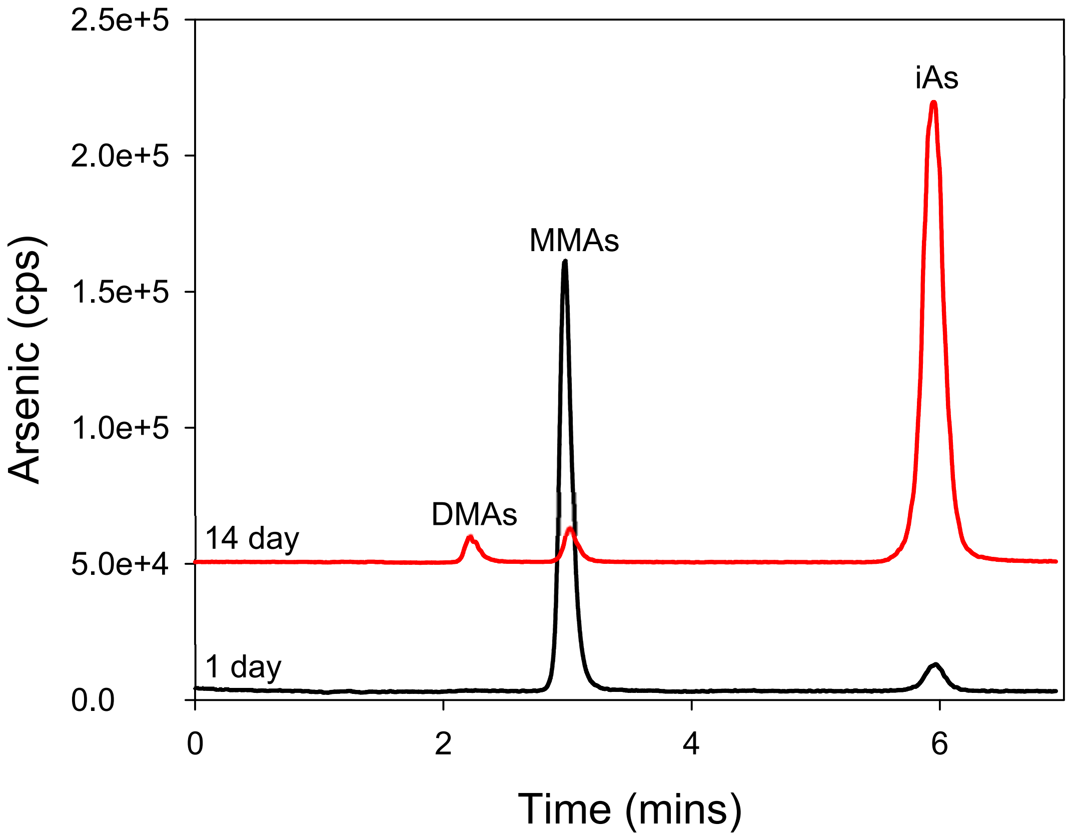

Fig. S9 Demethylation of MMAs in the enrichment culture of methanogens from CZ soil. Arsenic speciation by HPLC-ICP-MS of the culture medium on day 1 and day 14. 20 mM methanol and 2 μM MMAs were added to the enrichment culture.

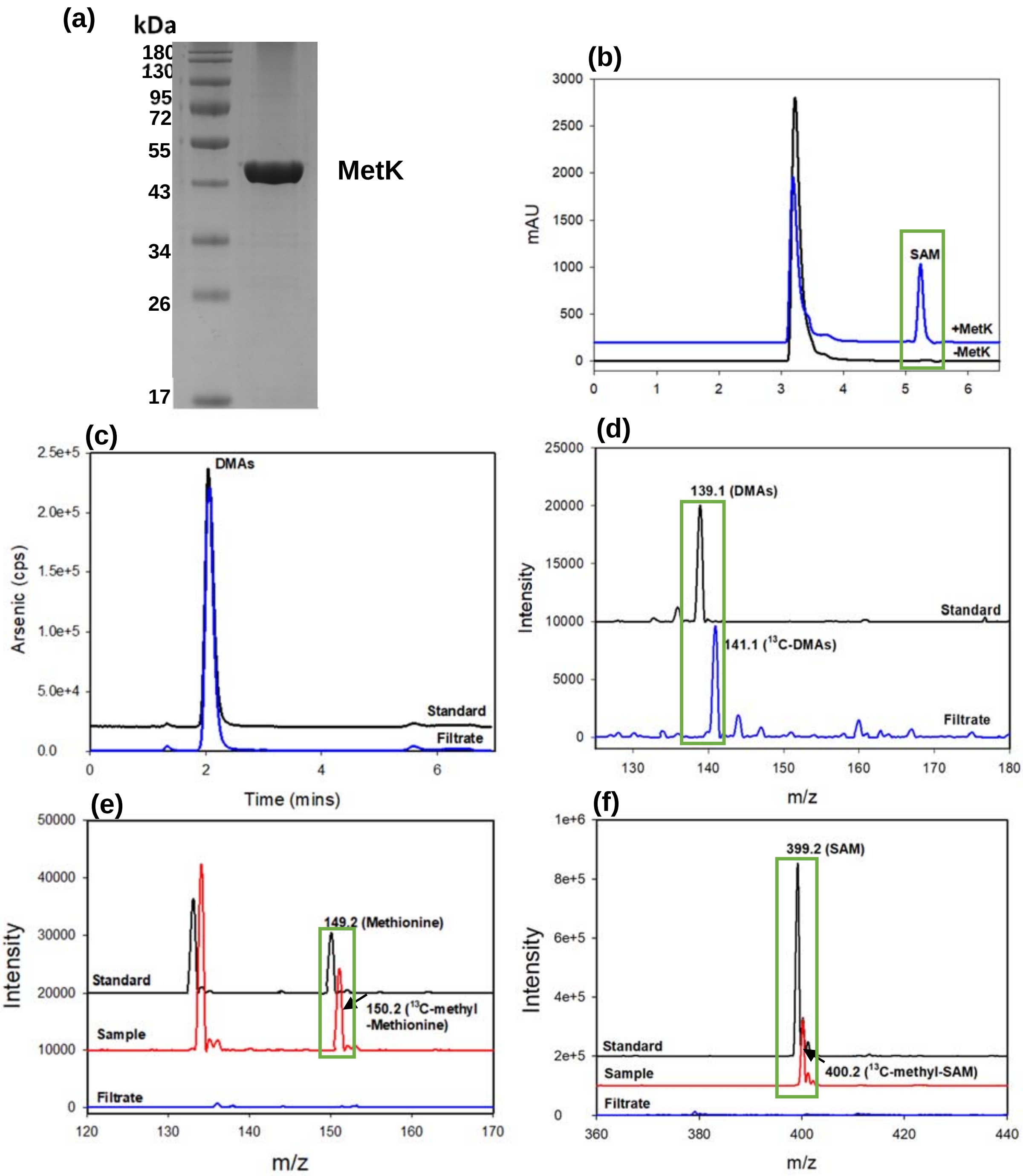

Fig. S10 Enzymatic synthesis and purification of ^{13}C-DMAs. (**a**) Purification of MetK was identified by SDS-PAGE. (**b**) Detection of ^{13}C-methyl-SAM in the reaction mixture by HPLC-UV. (**c**) Detection of ^{13}C-DMAs in the filtrate by HPLC-ICP-MS. (**d**) Detection of ^{13}C-DMAs in the filtrate by HPLC-ESI-MS/MS. (**e**) Detection of ^{13}C-methyl-Methionine in the reaction mixture (Sample) and the filtrate. (**f**) Detection of ^{13}C-methyl-SAM in the reaction mixture (Sample) and the filtrate.

Supplementary Tables

Table S1 The primers used in the present study

Primer No.	Name	Sequence [a]
P1	515F	GTGCCAGCMGCCGCGG
P2	907R	CCGTCAATTCMTTTRAGTTT
P3	Arch519F	CAGCCGCCGCGGTAA
P4	Arch915R	GTGCTCCCCCGCCAATTCCT
P5	DSR-F+	ACSCACTGGAAGCACGGCGG
P6	DSR-R	GTGGMRCCGTGCAKRTTGG
P7	mlas	GGTGGTGTMGGDTTCACMCARTA
P8	mcrA-rev	CGTTCATBGCGTAGTTVGGRTAGT
P9	arsM-309F	GYIWWNGGIVTNGAYATGA
P10	arsM-470R	ARRTTIAYIACRCARTTNS
P11	BlarsM-F	GGGAATTC**CATATG**ATGAGCGAATCAAATTTTCAGACAT
P12	BlarsM-R	CCG**CTCGAG**TTTTTTACCGCAAATCAGATAATAACC
P13	DmarsM-F	GGGAATTC**CATATG**ATGATAGACACTAAAGAAGAGATTAGAGACTT
P14	DmarsM-R	CCG**CTCGAG**TTTTCTTGCCTCGATAATATAAGAAGC
P15	DgarsM-F	GGGAATTC**CATATG**ATGAAAGACAAAGAAGATATCAGGGAAT
P16	DgarsM-R	CCG**CTCGAG**TTCCTTTACCGCCTCAATTATATAAGA
P17	metK-F	GGGAATTC**CATATG**ATGGCAAAACACCTTTTTACGTC
P18	metK-R	CCG**CTCGAG**CTTCAGACCGGCAGCATCG

[a] Bold and underlined letters represent enzyme restriction sites.

Table S2 The culture media used in the present study

	Composition
SRB media	NaCl 1.17 g L^{-1}, $MgCl_2 \cdot 6H_2O$ 0.4 g L^{-1}, KCl 0.3 g L^{-1}, $CaCl_2 \cdot 2H_2O$ 0.15 g L^{-1}, NH_4Cl 0.27 g L^{-1}, KH_2PO_4 0.20 g L^{-1}, Yeast extract 1 g L^{-1}, 20 mM acetate, $NaHCO_3$ 2 g L^{-1}, 20 mM Na_2SO_4. Trace element solution and Vitamin solution 1.
Methanogens media	$MgCl_2 \cdot 6H_2O$ 0.4 g L^{-1} NH_4Cl 0.1 g L^{-1}, $CaCl_2 \cdot 2H_2O$ 0.1g L^{-1}, KCl 0.5 g L^{-1}, KH_2PO_4 0.2 g L^{-1}, L-cysteine-HCl 0.3 g L^{-1}. 5 mM acetate. $NaHCO_3$ 2 g L^{-1}. Trace element solution and Vitamin solution 2.
Trace elements solution (1000×)	$FeCl_2 \cdot 4H_2O$ 2 g L^{-1}, $ZnCl_2 \cdot$0.07 g L^{-1}, $MnCl_2 \cdot 4H_2O$ 0.1 g L^{-1}, $CuCl_2 \cdot 2H_2O$ 0.002 g L^{-1}, $NiCl_2 \cdot 6H_2O$, 0.024 g L^{-1}, H_3BO_3 0.06 g L^{-1}, $CoCl_2 \cdot 6H_2O$ 0.19 g L^{-1}, Na_2MoO_4 0.036 g L^{-1}.
Vitamin solution 1 （100×）	Biotin 2 mg L^{-1}, Folic acid 2 mg L^{-1}, Pyridoixine-HCl 10 mg L^{-1}, Thiamine-HCl$\cdot 2H_2O$ 5 mg L^{-1}, Riboflavin 5 mg L^{-1}, Nicotinic acid 5 mg L^{-1}, D-Ca-pantothenate 5 mg L^{-1}, Vitamin B12 0.1 mg L^{-1}, p-Aminobenzoic acid 5 mg L^{-1}, Lipoic acid, 5 mg L^{-1}.
Vitamin solution 2 (1000×)	Aminobenzoic acid 0.04 g L^{-1}, Biotin 0.01 g L^{-1}, Lipoic acid 0.01 g L^{-1}, Vitamin B2 0.05 g L^{-1}, D-Ca-pantothenate 0.1 g L^{-1}, Vitamin B6 0.1 g L^{-1}, Folic acid 0.03 g L^{-1}, Nicotinic acid 0.05 g L^{-1}. Thiamine 0.01 g L^{-1}, Vitamin B12 0.05 g L^{-1}.

Table S3 The relative abundance (>1%) of core genera of bacteria in the enrichment cultures of SRB from CZ, QY and XY paddy soils. Data are means ± SE (*n* = *3*). Bold letters represent SRB.

Genera (CZ)	Relative abundance (>1%)	Genera (QY)	Relative abundance (>1%)	Genera (XY)	Relative abundance (>1%)
Clostridiaceae 1_uncultured	13.98±1.42	*Fonticella*	35.40±2.96	*Clostridium sensu stricto* 13	16.36±1.81
Epulopiscium	9.63±0.49	*Oscillibacter*	9.58±2.17	*Anaerocolumna*	10.74±1.83
Desulfotomaculum	6.70±0.27	*Clostridium sensu stricto* 1	5.13±1.26	*Fonticella*	10.66±0.61
Ruminococcaceae UCG-009	6.27±0.68	*Hydrogenoanaerobacterium*	4.78±0.26	*Lutispora*	7.44±1.10
Lutispora	5.94±0.92	*Caproiciproducens*	3.92±0.54	*Clostridium sensu stricto* 7	7.38±0.74
Sedimentibacter	5.42±0.44	*Anaerosporomusa*	3.53±0.25	***Terrisporobacter***	6.64±0.69
Terrisporobacter	4.07±0.17	*Anaerocolumna*	3.43±0.66	Ruminococcaceae UCG-010	5.83±0.79
Paraclostridium	4.05±0.07	*Ruminiclostridium* 9	3.25±0.13	*Proteiniborus*	4.80±0.87
Hydrogenoanaerobacterium	4.04±0.07	*Clostridium sensu stricto* 3	2.68±1.23	*Hydrogenoanaerobacterium*	4.79±0.32
Desulfovibrio	4.00±0.5	Veillonellaceae_uncultured	2.38±0.24	***Desulfovibrio***	3.51±0.50
Sporanaerobacter	3.61±0.34	Family XIII_uncultured	2.19±0.50	*Sedimentibacter*	2.60±0.46
Clostridium sensu stricto 13	3.40±0.36	*Rubeoparvulum*	1.97±0.52	***Desulfosporosinus***	2.14±0.16
Clostridium sensu stricto 7	3.31±0.31	*Sporanaerobacter*	1.94±0.50	*Sporanaerobacter*	2.04±0.15
Clostridium sensu stricto 1	2.73±0.53	*Clostridium sensu stricto* 7	1.88±0.34	Veillonellaceae_uncultured	1.62±0.29
Ruminiclostridium 9	2.48±0.15	*Clostridium sensu stricto* 13	1.72±0.44	Christensenellaceae R-7 group	1.45±0.20
Anaerotruncus	2.19±0.17	*Ruminococcaceae*_uncultured	1.65±0.17	BSV40_norank	1.37±0.15
Ruminococcaceae UCG-010	1.97±0.12	*Butyricicoccus*	1.34±0.12	*Clostridium sensu stricto* 1	1.27±0.26
BSV40_norank	1.70±0.10	*Clostridium sensu stricto* 14	1.31±0.48	*Lachnoclostridium* 5	1.22±0.26
Ruminiclostridium 1	1.26±0.10	Ruminococcaceae UCG-010	1.06±0.10	*Ruminiclostridium* 9	1.20±0.01
Christensenellaceae R-7 group	1.14±0.06	*Christensenellaceae*_uncultured	1.04±0.05	*Anaerovorax*	1.01±0.02
SRB2_norank	1.12±0.04	***Desulfosporosinus***	0.09±0.06		
Romboutsia	1.10±0.15	***Terrisporobacter***	0.26±0.00		

Table S4 Mass balance and distribution of arsenic species in the soil solution and solid phases, and volatile arsenic the headspace in the incubation of CZ, QY and XY paddy soils amended with DMAs (0.8 µmol per bottle of 20 g soil). Data are means ± SE (*n* = *3*).

Soil	Time (Day)	Solution			Solid phase			Volatile As (ng)	Sum of As species (µmol)
		DMAs (µmol)	MMAs (µmol)	iAs (µmol)	DMAs (µmol)	MMAs (µmol)	iAs (µmol)		
CZ	1	0.20±0.01	0	0	0.69±0.02	0	4.66±0.31	ND [a]	5.45±0.28
	30	0.01±0.00	0.12±0.01	0.25±0.01	0.02±0.01	0.18±0.01	4.93±0.97	ND	5.54±0.80
QY	1	0.02±0.01	0	0	0.70±0.02	0	2.35±0.29	ND	3.07±0.25
	30	0.005±0.00	0.18±0.3	0.06±0.00	0.03±0.00	0.21±0.03	2.89±0.01	ND	3.30±0.17
XY	1	0.12±0.04	0	0	0.62±0.03	0	0.50±0.05	ND	1.24±0.01
	30	0	0.005±0.00	0.002±0.00	0.06±0.02	0.19±0.08	1.15±0.00	ND	1.41±0.01

[a] ND, not detectable.

Table S5 The relative abundance (>1%) of core genera of archaea in the enrichment cultures of methanogens from CZ, QY and XY paddy soils. Data are means ± SE (*n* = *3*). Bold letters represent methanogens.

**Genera (CZ)**	**Relative abundance (>1%)**	**Genera (QY)**	**Relative abundance (>1%)**	**Genera (XY)**	**Relative abundance (>1%)**
Soil Crenarchaeotic Group(SCG)_norank	65.38±2.92	***Methanobacterium***	32.60±9.36	***Methanobacterium***	52.32±5.68
Bathyarchaeota _norank	19.67±2.00	Bathyarchaeota_norank	21.47±3.38	Bathyarchaeota_norank	24.76±2.64
Methanobacterium	8.42±1.28	Woesearchaeota (DHVEG-6)_norank	17.43±8.15	***Methanosarcina***	19.57±7.43
Woesearchaeota (DHVEG-6)_norank	1.79±0.53	***Methanosarcina***	8.73±1.26	***Methanomassiliicoccus***	1.63±1.10
CCA47_norank	1.44±0.37	***Methanomassiliicoccus***	8.34±1.05		
Methanosarcina	1.27±0.17	Soil Crenarchaeotic Group(SCG)_norank	5.14±0.40		
Methanomassiliicoccus	1.13±0.18	***Methanocella***	2.41±0.49		
